# Clinicopathologic study of invasive micropapillary carcinoma of the breast

**DOI:** 10.18632/oncotarget.16405

**Published:** 2017-03-21

**Authors:** Shen-li Tang, Ji-qiao Yang, Zheng-gui Du, Qiu-wen Tan, Yu-ting Zhou, Di Zhang, Qing Lv

**Affiliations:** ^1^ Department of Breast Surgery, West China Hospital/West China School of Medicine, Sichuan University, 610041, P.R. China; ^2^ Laboratory of Breast Disease, West China Hospital/West China School of Medicine, Sichuan University, 610041, P.R. China

**Keywords:** breast cancer, invasive micropapillary carcinoma, locoregional recurrence, prognostic factors, survival

## Abstract

Invasive micropapillary carcinoma (IMPC) is a rare subtype of breast carcinoma. It is presumed to be more aggressive than invasive ductal carcinoma (IDC), though it is uncertain whether the prognoses of IMPC and IDC differ. In this retrospective study, we compared the clinicopathologic characteristics and survival between 170 female patients with IMPC (pure or mixed with IDC) and 728 with pure IDC. The IMPC patients had higher clinical stages and histologic grades, higher incidences of lymphovascular invasion and axillary lymph node extracapsular extension, and a higher degree of lymph node involvement than IDC patients. Moreover, IMPC was associated with increases in estrogen receptor (ER) and progesterone receptor (PR) positivity and HER-2 overexpression. Although locoregional recurrence-free survival (LRRFS) and recurrence-free survival (RFS) were poorer in IMPC patients than IDC patients, overall survival and distant metastasis survival did not differ between the two groups. Multivariate analysis revealed that IMPC was an independent prognostic factor for LRRFS in breast cancer, and IMPC patients had poorer clinicopathologic characteristics and poorer RFS and LRRFS than IDC patients. We therefore suggest that to improve treatment decisions, patients with breast carcinoma be tested for the presence of this specific subtype.

## INTRODUCTION

Invasive micropapillary carcinoma (IMPC) of the breast, which was first defined by Siriaunkgul and Tavassoli, is characterized by micropapillae surrounded by empty stromal spaces [1, 2]. Since then, several researchers have examined the clinical outcomes and pathologic features of IMPC [3, 4]. In the 2003 World Health Organization (WHO) guidelines for histologic classification of tumors of the breast [5], IMPC was listed as a rare subtype of invasive breast carcinoma, accounting for approximately 2% to 8% of all breast cancers [6–8].

The relatively low incidence of IMPC makes it difficult to directly compare its clinical outcomes and pathologic features to invasive ductal carcinoma (IDC). Therefore, IMPC patients are treated using standard IDC treatments. However, IMPC is more likely to have aggressive characteristics, such as high incidences of axillary lymph node (ALN) metastasis and local recurrence, compared to IDC [9, 10]. Although this would be expected to result in poorer outcomes for IMPC patients compared to IDC patients, reports have indicated that they have similar prognoses [6, 11]. However, differences in the prognoses of IMPC and IDC require further investigation, and identifying the features that distinguish IMPC from IDC would help to improve disease management for IMPC patients.

In this retrospective study, we compared clinicopathologic characteristics and clinical outcomes of IPMC and IDC patients to gather information that might help to optimize management strategies for and improve the outcomes of IMPC patients.

## RESULTS

### Patients

Among the 3,693 patients diagnosed with invasive breast cancer at our institution between January 2000 and April 2016 who were included in the database, we identified 170 IMPC patients (4.6%) who received standard curative treatment without neoadjuvant chemotherapy. These patients were compared to randomly selected control IDC patients who were diagnosed during the same period. Ultimately, a total of 898 patients (170 IMPC and 728 IDC) were enrolled in this study. Among the 170 IMPC patients, 154 (90.6%) had a 10% to 90% micropapillary growth pattern with IDC components, while 16 (9.4%) cases had a more than 90% micropapillary growth pattern.

The mean age at diagnosis of both IMPC and IDC patients was 48 years (range, 23-77 years for IMPC and 23-88 years for IDC patients). The median follow-up duration was 40 (range, 5-180) months for IMPC patients and 60 (range, 3-191) months for IDC groups. Clinicopathologic characteristics of the IDC and IMPC patients are summarized in Table 1.

**Table 1:** Clinical and pathologic characteristics of IMPC and IDC patients

Characteristic	Parameter	IMPC(n=170)	IDC (n=728)	*P*
**Clinical**				
Age (years)	median	48.78	48.45	0.650
	range	23-77	23-88	
Mastectomy	Total	164 (97.0%)	663 (92.6%)	0.036
	Partial	5 (3.0%)	53 (7.4%)	
	Unknown	1	12	
Extent of lymph node surgery	No surgery	0	5(0.7%)	0.072
	SLNB	4 (2.4%)	16 (2.2%)	
	ALNB	160 (94.1%)	686 (95.7%)	
	SLNB+ALNB	6(3.5%)	10 (1.4%)	
	Unknown	0	11	
Chemotherapy	Used	157 (92.4%)	695 (95.5%)	0.097
	Not used	13 (7.6%)	33 (4.5%)	
Radiotherapy	Used	73 (42.9%)	286 (39.3%)	0.381
	Not used	97 (57.1%)	442 (60.7%)	
Hormone therapy	Used	135 (86.5%)	472 (64.8%)	**<0.001**
	Not used	21 (13.5%)	256 (35.2%)	
	Unknown	14	0	
**Pathologic**				
Tumor stage	T1	48 (31.6%)	224 (34.0%)	0.654
	T2	90 (59.2%)	377 (57.3%)	
	T3	8 (5.3%)	41(6.2%)	
	T4	6 (3.9%)	16 (2.4%)	
	Unknown	18	70	
Nodal stage	N0	59 (35.1%)	349 (48.5%)	**<0.001**
	N1	43 (25.6%)	210 (29.2%)	
	N2	31 (18.5%)	86 (11.9%)	
	N3	35 (20.8%)	75 (10.4%)	
	Unknown	2	8	
AJCC stage	1	24 (16.3%)	133 (20.5%)	**<0.001**
	2	53 (36.1%)	349 (53.7%)	
	3	70 (47.6%)	168 (25.8%)	
	Unknown	23	78	
Histologic grade	1	3 (2.4%)	17 (3.4%)	**0.036**
	2	56 (44.4%)	162 (32.1%)	
	3	67 (53.2%)	326 (64.6%)	
	Unknown	44	223	
Positive lymph nodes	None	60 (35.7%)	315 (46.8%)	**<0.001**
	1-3	44 (26.2%)	205 (30.5%)	
	4 or more	64 (38.1%)	153 (22.7%)	
	Unknown	2	55	
LVI	Positive	25 (14.7%)	1 (0.1%)	**<0.001**
	Negative	145 (85.3%)	727 (99.9%)	
BVI	Positive	3 (1.8%)	16 (2.2%)	0.784
	Negative	167 (98.2%)	712 (97.8%)	
ECE	Positive	12 (7.1%)	8 (1.1%)	**<0.001**
	Negative	158 (92.9%)	720 (98.9%)	
**Immunohistochemical**				
Estrogen receptor	Positive	142 (83.5%)	471 (65.7%)	**<0.001**
	Negative	28 (16.5%)	246 (34.3%)	
	Unknown	0	11	
Progesterone receptor	Positive	133 (78.2%)	464 (64.7%)	**0.001**
	Negative	37 (21.8%)	253 (35.3%)	
	Unknown	0	11	
HER2	Negative	93 (66.0%)	561 (85.6%)	**<0.001**
	Positive	48 (34.0%)	94 (14.4%)	
	Unknown	29	73	
Ki-67	<20%	55 (32.9%)	164 (27.9%)	0.210
	≥20%	112 (67.1%)	423 (72.1%)	
	Unknown	3	141	
Subtype	ER/PR^+^HER2^−^	91 (64.5%)	411 (63.5%)	**<0.001**
	ER/PR^+^HER2^+^	30 (21.3%)	54 (8.3%)	
	ER/PR^−^HER2^+^	18 (12.8%)	41 (6.3%)	
	Triple negative	2 (1.4%)	141 (21.8%)	
	unknown	29	81	

IMPC: invasive micropapillary breast cancer

IDC: invasive ductal carcinoma not otherwise specified

ALND: axillary lymph node dissection

SLNB: sentinel lymph node biopsy

AJCC: American Joint Committee On Cancer

LVI: lymphovascular invasion

BVI: blood vessel invasion

ECE: axillary lymph node extracapsular extension

HER2: human epidermal growth factor receptor-2

Ki-67: cell proliferation antigen protein

### Treatment

Total mastectomy was conducted more frequently in IMPC cases (164, 97.0%) compared to IDC cases (663, 92.6%) (*p*=0.036). Every patient who received BSC underwent radiotherapy. IMPC and IDC patients did not differ in the extent of lymph node surgery or in the conditions of systemic chemotherapy and postoperative radiation therapy. Hormone therapy (tamoxifen in most cases) was administered to 607 patients (67.6%) who were positive for hormone receptor expression, of whom 135 were IMPC patients and 472 were IDC patients (*p*<0.001).

### Clinicopathologic and IHC characteristics in IMPC and IDC

In the overall cohort, the T2 stage was dominant in both IMPC and IDC patients, and there were no differences in pT distribution between IMPC and IDC patients (*p*=0.654). In contrast, IMPC patients had higher pN stages than IDC patients (*p*<0.001). Furthermore, a higher proportion of IMPC patients (70, 41.2%) had stage III disease as identified by pathology compared to IDC patients (168, 25.8%) (*p*<0.001). Thus, IMPC patients had higher clinical stages and histological grades than IDC patients (*p*<0.001).

The number of metastatic axillary lymph nodes identified in patients ranged from 0 to 56 (mean, 3.0). IMPC patients were more likely than IDC patients to have 4 or more metastatic axillary lymph nodes (38.1%*vs*. 22.7%, *p*<0.001). Comparisons of the clinical and pathologic features of the IMPC and IDC cases revealed that IMPC patients had higher rates of ECE (7.1% *vs*. 1.1%, *p*<0.001) and LVI (14.7% *vs*. 0.1%,*p*<0.001); incidences of blood vessel invasion (BVI) did not differ between the two groups.

In immunohistochemical experiments, ER-positive (83.5%*vs*. 65.7%, *p*<0.001) and PR-positive (78.2%*vs*. 64.7%, *p*=0.001) tumors were detected more frequently in the IMPC group. In addition, HER2 overexpression was detected more often in the IMPC group (34.0% vs 14.4%, *p*<0.001). However, the proportion of tumors with high Ki-67 indexes did not differ between the two groups (83.3%*vs*. 78.6%). Furthermore, IMPC and IDC patients also differed with regard to molecular subtype as indicated by combined ER/PR/HER2 status (*p*<0.001); the incidence of triple negative breast cancer (TNBC) was lower in IMPC (2, 1.4%) than in IDC (141, 21.8%) patients, while luminal A, luminal B, and HER-2 overexpression occurred more frequently in IMPC than in IDC patients.

### Differences in survival between IMPC and IDC

Clinical follow-up information was collected over periods ranging from 3 to 191 months (mean, 53.5). The median follow-up period was 40 months for IMPC patients and 60 months for IDC patients (range, 5-180 months and 3-191 months, respectively).

Overall, 73 patients (8.1%) died of disease-related causes between 3 and 191.0 months after treatment (mean, 53.0). Eight patients (4.7%) with IMPC died between 5.9 and 138 months after treatment (mean, 38.5), whereas 65 patients (8.9%) with IDC died between 3 and 191.0 months after treatment (mean, 58.3). Overall survival did not differ between IMPC and IDC patients (*p*=0.070).

Overall, LLR was detected in 33 patients during follow-ups (3.6%). The mean durations of LRR in IMPC and IDC patients were 38.5 and 58.3 months, respectively. LRR was detected 4.2% more often (*p*=0.009) in IMPC patients compared to IDC patients. Additionally, LRR occurred approximately 20 months earlier in IMPC patients than in IDC patients. All patients with LRR received RT targeting the remnant breast/chest wall and supraclavicular fossa. Overall, 102 patients (10.5%), including 14 IMPC and 88 IDC patients, developed DM. The mean durations of DM for IMPC and IDC patients were 38.7 and 57.4 months, respectively.

### Prognostic markers for IMPC

**Figure 1:**
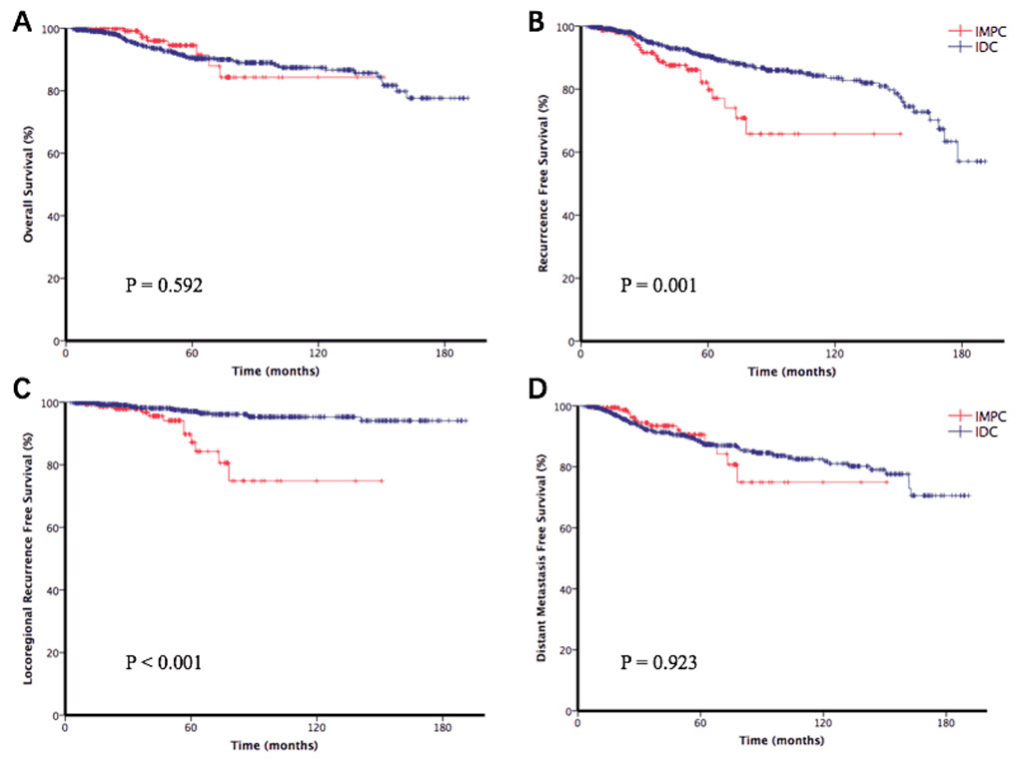
Survival analysis for overall survival (OS) rate **(A)**, recurrence-free survival (RFS) rate **(B)**, loco-regional recurrence-free survival (LRRFS) rate **(C)**, and distant metastasis-free survival (DMFS) rate **(D)** in the IMPC and IDC patients.

The 5-year and 10-year OS rates were 94.5% and 84.3% for IMPC patients and 90.6% and 87.4% for IDC patients, respectively; OS rates did not differ between IMPC and IDC patients (Figure 1A, log-rank=0.592). The RFS rate (Figure 1B, log-rank=0.001) and the LRRFS rate (Figure 1C, log-rank<0.001) were lower in IMPC patients than in IDC patients. The 5-year DMFS rate (Figure 1D, log-rank= 0.923) did not differ between the two groups (IMPC 91.5%, IDC 88.3%).

Differences in OS, RFS, LRRFS, and DMFS in the overall patient cohort depending on possible prognostic factors are shown in Table 2. To confirm the effects of these factors on RFS and LRRFS, we performed multivariate analysis. The results of multivariate analysis were shown in Table 3 and Table 4, which indicated that pN stage (*p*=0.004) was a powerful prognostic factor for RFS, while the presence of an IMPC component (*p*=0.004) was a powerful prognostic factor for LRRFS. The limited simple size may have prevented the detection of other potentially significant prognostic factors.

**Table 2:** Prognostic factors-univariate analysis

Characteristic	Parameter	No of pts (%)	P-value			
			OS	RFS	LRRFS	DMFS
Age	>35	76(8.5%)	0.496	0.455	0.479	0.365
	≤35	822(91.5%)				
Surgery type	total	827(92.1%)	0.654	0.563	0.913	0.253
	partial	58(6.5%)				
	unknown	13(1.4%)				
Axillary LN evaluation	none	5(0.6%)	0.991	0.461	0.937	0.44
	SLNB	20(2.2%)				
	ALNB	846(94.2%)				
	SLNB+ALNB	16(1.8%)				
	Unknown	11(1.2%)				
Positive lymph nodes	none	375(41.8%)	**<0.0001**	**<0.0001**	**<0.0001**	**<0.0001**
	1-3	249(27.7%)				
	4 or more	217(24.2%)				
	Unknown	57(6.3%)				
pT stage	1	272(30.3%)	**<0.0001**	0.201	**0.014**	**<0.0001**
	2	467(52.0%)				
	3	49(5.5%)				
	4	22(2.4%)				
	Unknown	88(9.8%)				
pN stage	0	408(45.4%)	**<0.0001**	**<0.0001**	**0.002**	**<0.0001**
	1	253(28.2%)				
	2	117(13.1%)				
	3	110(12.2)				
	Unknown	10(1.1%)				
LVI	Positive	26(2.9%)	0.042	0.026	0.084	0.043
	Negative	872(97.1%)				
BVI	Positive	19(2.1%)	**0.001**	0.208	0.506	0.007
	Negative	879(97.9%)				
ECE	Positive	20(2.2%)	0.853	0.26	0.038	0.622
	Negative	878(97.8%)				
ER	Positive	613(69.3%)	**0.009**	0.6	0.853	0.418
	Negative	274(30.5%)				
	Unknown	11(1.2%)				
PR	Positive	597(66.5%)	**0.005**	0.561	0.49	0.112
	Negative	290(32.2%)				
	Unknown	11(1.3%)				
HER2	Negative	654(72.8%)	0.614	0.059	0.468	0.717
	Positive	142(15.8%)				
	Unknown	102(11.4%)				
Ki-67	<20%	219(24.4%)	**0.014**	0.487	0.540	0.804
	≥ 20%	535(59.6%)				
	Unknown	144(16.0%)				
Subtype	ER/PR+HER2-	502(55.9%)	0.215	0.848	0.842	0.378
	ER/PR+HER2+	84(9.4%)				
	ER/PR-HER2+	59(6.6%)				
	Triple negative	143(15.9%)				
	Unknown	110(12.2%)				
Histologic grade	I	20(2.2%)	0.079	0.853	0.745	0.757
	II	218(24.3%)				
	III	393(43.8%)				
	Unknown	267(29.7%)				
Chemotherapy	Used	852(94.9%)	0.325	0.848	0.313	0.005
	Not used	46(5.1%)				
Radiotherapy	Used	359(40.0%)	0.325	**0.003**	**0.002**	**0.013**
	Not used	539(60.0%)				
Hormone therapy	Used	607(67.6%)	**<0.0001**	0.11	0.81	**0.007**
	Not used	277(30.8%)				
	Unknown	14(1.6%)				
IMPC	Positive	170(18.9%)	0.592	**0.003**	**0.002**	0.923
	negative	728(81.1%)				

OS: overall survival

RFS: recurrence-free survival

LRRFS: loco-regional recurrence-free survival

DMFS: distant metastasis-free survival

pT stage: pathologic T stage

pN stage: pathologic N stage

ER: estrogen receptor

PR: progestrone receptor

**Table 3:** Prognostic factors of recurrence-multivariate analysis

Variables	B	p-value	HR	95%CI
pathologic T	0.153	0.33	1.166	0.865-1.588
pathologic N	0.475	**0.002**	1.609	1.187-2.180
positive LN	0.165	0.436	1.18	0.799-1.788
LVI	0.406	0.463	1.501	0.507-4.439
radiotherapy	−0.014	0.955	0.986	0.605-1.606
IMPC negative	−0.344	0.24	0.709	0.399-1.259

**Table 4:** Prognostic factors of local and regional recurrence-multivariate analysis

Variables	B	p-value	HR	95%CI
pathologic N	0.097	0.706	1.102	0.664-1.829
positive LN	0.619	0.092	1.858	0.904-3.818
ECE	0.536	0.49	1.71	0.373-7.838
radiotherapy	−0.575	0.217	0.562	0.225-1.404
IMPC negative	−1.155	**0.004**	0.315	0.143-0.693

LN: lymph node

## DISCUSSION

Breast cancer is now the primary cause of death among women in China [12]. IMPC is a rare and unique pathologic subtype of breast carcinoma. Since Fisher *et al*. [13] first described it, various reports have further characterized IMPCs [9, 14, 15]. This rare variant of invasive breast cancer may occur either alone or in combination with other histologic types of breast cancer [8, 16]. Previous studies reported that most patients had mixed IMPC [17, 18], which is consistent with our findings here that 154 (90.6%) patients had a 10% to 90% micropapillary growth pattern with other components.

In this study, we enrolled patients that had IMPC histology both alone (“pure IPMC”) or mixed with IDC, since there is no established IMPC component proportion criteria when diagnosing IMPC [19]. Moreover, in a genomic analysis comparing 12 patients with pure IPMC and 24 hormone receptor- and grade-matched IDC patients, Marchio *et al*. demonstrated that IMPC had distinct histological features and molecular genetic profiles compared to IDC [20, 21]. Furthermore, their comparison of 24 pure and 40 mixed IMPCs suggested that mixed IMPCs were more closely related to pure IMPCs than to IDCs. Thus, micropapillary differentiation in breast cancer may be indicative of distinct pathological and genetic characteristics, regardless of the IMPC component proportion.

Previous studies indicate that IMPC components are strongly associated with extensive LVI and LN metastasis [22–24]. Recently, Li *et al*. [25] found that LN metastases and LVI occur at higher rates in mixed IMPC patients than in IDC patients; Shi *et al*. obtained similar results [17]. In this study, we confirmed that LN metastases and LVI occurred more frequently in IMPC patients, who also had higher histologic grades and ECE; these results suggest that IMPC may be associated with a higher risk of tumor recurrence and distant metastasis.

Although ER- and PR-positive status is often correlated with improved prognosis in breast cancer patients, IMPC appears to be an exception [26]. IMPC patients are more likely to be positive for ER and PR expression than patients with other types of breast cancer [6, 27]. Consistent with previous studies [6, 11, 18, 23, 28–30], we found that more IMPC patients were positive for ER and PR staining compared to IDC patients; however, the Ki-67 index did not differ between the groups. In addition, HER2 overexpression occurred more frequently in the IMPC group than in the IDC group, which is inconsistent with the results of a previous study. Increased HER2 expression in IMPC may contribute to the highly invasive characteristics of this tumor type.

Although IMPC is associated with advanced and aggressive clinicopathologic features, it remains unclear whether clinical outcomes for IMPC differ from those of IDC. In a study using the US National Cancer Institute's Surveillance, Epidemiology, and End Results (SEER) database, Chen *et al*. found that OS was similar in IDC and IMPC patients, even though lymph node metastasis was more common in the latter [6]. OS was also similar for IDC and IMPC patients in Jeong Yu *et al*.'s study [28], although LVI rate, nuclear grade, RFS rate, and LRRFS rate were higher in the IMPC group than in the IDC group. In contrast, Chen *et al*. [23] found that LVI was higher and 5-year OS was poorer in IMPC patients compared to IDC patients. Similarly, Shi *et al*. [17] found that OS and RFS were poorer in the IMPC group than in the IDC group. Our present results confirmed that RFS and LRRFS rates were worse in IMPC patients than in IDC patients. Additionally, LRR occurred approximately 20 months earlier in IMPC patients than in IDC patients. However, OS and DMFS rates did not differ between IMPC and IDC patients, perhaps due to the short follow-up period and limited sample size.

Our current findings also provide some useful information about recurrence of IMPC. Yu *et al*. [28] found that the LRR rate was higher in IMPC than IDC and therefore suggested more thorough examinations and more aggressive therapies for IMPC patients, including larger surgical margins, more extensive axillary LN dissection, and higher doses of and/or axillary RT. Similarly, we found that LRR rates were higher in IMPC than in IDC. Additionally, and consistent with previous reports that the presence of IMPC is strongly associated with higher recurrence rates [23], we found that the presence of IMPC was a significant prognostic factor for LRRFS. Thorough loco-regional examinations and aggressive therapies should therefore be used for IMPC patients to accurately evaluate local tumor conditions and to provide appropriate and standardized treatments that minimize local recurrence.

Pathological characteristics associated with highly invasive cancer, such as HER-2+ status and higher histologic grades, LVI, ECE, and lymph node involvement, suggest that IMPC tumors are distinct and heterogeneous. It is possible that IMPC patients do not differ in OS and DMFS because they receive endocrine therapy much more often than IDC patients. However, RFS and LRRFS rates are worse in IMPC patients, indicating that their prognoses may still be relatively poor and that improvements in treatment are needed. Thus, breast carcinoma specimens should be carefully examined for the presence of IMPC to ensure that the disease is managed properly.

Some limitations of this study should be considered when interpreting the results. First, selection biases were unavoidable due to our use of retrospective data. Second, the pathologic and/or IHC data were based on institutional reports rather than central pathologic review, although diagnoses were made by highly experienced breast cancer experts at qualified academic hospitals. Furthermore, it is possible that IDC group patients, who were randomly selected using SPSS, may be not representative of IDC patients overall. Finally, Ki-67 staining and ER\PR-positive percentage were not included in the subtype analysis due to a lack of sufficiently detailed data. Despite these limitations, our study provides a relatively comprehensive examination of the clinicopathologic and immunohistochemical characteristics of IMPC, the impact of adjuvant systemic therapy, and long-term patient survival.

In conclusion, our data confirm that IMPC is characterized by aggressive clinicopathologic features, and that IMPC patients have poorer RFS and LRRFS than IDC patients. Additionally, the presence of IMPC was an independent prognostic factor for LRRFS in breast cancer. Thus, breast carcinoma specimens should be tested for the presence of this specific tumor subtype, and therapeutic plans should be adjusted according to the results of those tests. Additional studies, especially large-scale prospective studies that also examine genomic expression, are needed to help establish more optimal management guidelines for this uncommon histological variant of breast carcinoma.

## MATERIALS AND METHODS

This retrospective analysis of anonymous data was approved by the West China Hospital research ethics committee. Because the study was retrospective, signed informed consent from patients was not required.

### Patient selection

We retrospectively recruited patients with breast cancer who were diagnosed and treated at West China Hospital of Sichuan University between January 2000 and April 2016. A total of 3963 patients were added to the database during this period. The following patient data were recorded: clinicopathologic features, follow up information, and survival.

Women with breast cancer who had pathologically confirmed IMPC and received standard treatment were considered for this study, regardless of the extent of the disease or the type of surgery performed. IDC patients who underwent surgery during the same time period were randomly selected for comparison. Patients included in the database were excluded from this analysis for the following reasons: (1) male patients, (2) presence of *in*-*situ* lesion, (3) curative resection was not conducted, (4) neo-adjuvant chemotherapy was administered or distant metastasis was confirmed before surgery, (5) survival data were unavailable. Patients with nonsynchronous contralateral tumors or tumor recurrence were counted only once for survival analyses, and the time at which the first tumor occurred was considered the beginning of the follow-up period.

The following features of IDC and IMPC patients were extracted from the database for analysis: age at diagnosis, pathologic T (pT) stage, pathologic N (pN) stage, degree of metastatic lymph nodes, presence of lymphovascular invasion (LVI), presence of blood vessel invasion (BVI), axillary lymph node extracapsular extension (ECE), therapeutic intervention (surgery, extent of lymph node surgery, hormone therapy, chemotherapy and radiation therapy), hormone receptor status, human epidermal growth factor receptor-2 (HER2) and Ki-67 status of the tumor, molecular subtype, and clinical outcome.

### Immunohistochemical criteria

In this study, tumor samples were considered positive for ER and PR expression if nuclear stating was observed in at least 1% of the tumor cells, and high Ki-67 expression was defined by the presence of immunostaining in more than 20% tumor cells, according to the recommendations of the 14th St. Gallen International Breast Cancer Conference [31]. HER2 staining was scored and categorized as follows: 0 (no immunostaining) and 1+ (immunostaining in ≤ 10% of tumor cells) were considered negative; 2+ (weak or incomplete membrane immunostaining in > 10% of tumor cells and complete membrane immunostaining in ≤ 10% of tumor cells) was considered inconclusive; and 3+ (strong, complete membrane immunostaining in > 10% of tumor cells) was considered positive. Fluorescence *insitu* hybridization (FISH) was used to confirm the presence of HER2 in samples with immunohistochemistry scores of 2+ according to the American Society of Clinical Oncology/College of American Pathologists (ASCO/CAP) clinical practice guidelines [32].

### Treatment

Patients underwent either modified radical mastectomy or breast conserving surgery (BCS). For patients in both groups, re-excision was conducted if the margins were not devoid of malignancy after the initial procedure. All clinically node-positive patients underwent axillary lymph node dissection (ALND), while sentinel lymph node biopsy (SLNB) was also conducted for negative patients beginning in 2010. Usually, level I and II lymph nodes were removed during ALND; if malignancy was suspected in level II and/or III lymph nodes, level III lymph nodes were also removed.

Patients in both groups who were treated with modified radical mastectomy and had primary tumors larger than 5 cm and/or involvement of ≥4 axillary lymph nodes (ALNs) received postoperative radiation therapy (RT). All patients who were treated with BCS also underwent postoperative radiotherapy. Patients received adjuvant chemotherapy after surgery.

### Follow-up

Follow-up data in the database was collected semiannually over the telephone and in routine follow-ups at clinics. All recurrences were diagnosed by either clinical examination or imaging. Locoregional recurrence (LRR) was defined as the appearance of local or regional tumors in any of the following places: ipsilateral breast, chest wall, axilla, internal mammary, ipsilateral supraclavicular area, or infra-clavicular area. Distant metastasis (DM) was defined as metastases to other sites.

### Statistical analysis

Clinicopathological analyses were performed using McNemar's test or the generalized McNemar's test, chi-squared tests, and Fisher's exact tests. The Kaplan–Meier curves were used to estimate survival, and the log-rank test was used to compare differences between tumor subtypes. The COX Proportional Hazards Model was used to conduct univariate and multivariate survival analyses. Results were considered significant at *p*<0.05 in all statistical tests, and all *p* values were 2-sided. All statistical analyses were performed using SPSS 19.0 statistical software (SPSS®, Chicago, Illinois, USA).
